# Ozone Environmental Pollution: Relationship between the Intestine and Neurodegenerative Diseases

**DOI:** 10.3390/antiox12071323

**Published:** 2023-06-22

**Authors:** Selva Rivas-Arancibia, Alfredo Miranda-Martínez, Erika Rodríguez-Martínez, Eduardo Hernández-Orozco, Marlen Valdés-Fuentes, Roberto De la Rosa-Sierra

**Affiliations:** Departamento de Fisiología, Facultad de Medicina, Universidad Nacional Autónoma de México, Mexico City 04510, Mexicoana_ery@yahoo.com.mx (E.R.-M.); eduardo.her.oro@gmail.com (E.H.-O.); marlen_valdez@yahoo.com.mx (M.V.-F.); delarosabeto_1987@comunidad.unam.mx (R.D.l.R.-S.)

**Keywords:** ozone, oxidative stress, bowel, brain, degenerative disease

## Abstract

Repeated exposure to environmental ozone causes a chronic state of oxidative stress. This state is present in chronic degenerative diseases and induces a loss of control of the inflammatory response. Redox system dysfunction and failures in control of inflammatory responses are involved in a vicious circle that maintains and increases the degenerative process. The intestine also responds to secondary reactive species formed by exposure to ozone doses, generating noxious stimuli that increase degenerative damage. This review aims to elucidate how environmental pollution, mainly by ozone, induces a state of chronic oxidative stress with the loss of regulation of the inflammatory response, both in the intestine and in the brain, where the functionality of both structures is altered and plays a determining role in some neurodegenerative and chronic degenerative diseases. For this purpose, we searched for information on sites such as the Cochrane Library Database, PubMed, Scopus, and Medscape. Reviewing the data published, we can conclude that environmental pollutants are a severe health problem. Ozone pollution has different pathways of action, both molecular and systemic, and participates in neurodegenerative diseases such as Parkinson’s and Alzheimer’s disease as well in bowel diseases as Inflammatory Bowel Disease, Crohn’s Disease, and Irritable Bowel Syndrome.

## 1. Environmental Pollution and Disease

Environmental pollution is one of the most urgent public health problems to solve. The World Health Organization (WHO) estimates that 4.2 million deaths per year can be attributed to air pollution, of which 237,000 deaths would be among children under six [1]. The deterioration of the environment due to the presence of materials and compounds such as carbon dioxide (CO_2_), carbon monoxide (CO), nitrogen oxide (NOx), sulfur dioxide (SO_2_), volatile organic compounds (VOCs), nitrogen dioxide (NO_2_), and ozone (O_3_) in amounts more significant than natural ones are an anthropogenic phenomenon [2]. The increase in these compounds is correlated with the rise in chronic degenerative diseases. Of the wide variety of pollutants that affect the quality of life of organisms, particulate matter (PM), specific microparticles with an aerodynamic diameter of less than 2.5 µm (PM_2.5_) and particles equal or less than 10 µm (PM_10_), stands out. Epidemiological studies have shown that the fraction of fine particles of PM_2.5_ has a more significant impact on health than PM_10_ [3]. PM_2.5_ is generated mainly in combustion processes, which produce carbon particles that may contain nitrates, sulfates, polycyclic aromatic hydrocarbons, and reactive metals such as iron, copper, nickel, zinc, and vanadium within their structure [4]. The physicochemical properties of the particles, such as size, structure, chemical composition, reactivity, and solubility, as well as the ability to enter the cell, determine their impact on health and the mechanisms by which PM induces this variety of effects [5]. It is estimated that O_3_ could be responsible for almost 17,000 premature deaths in Europe each year [6]. In addition, tropospheric ozone, as well as affecting health, has adverse effects on ecosystems, particularly forests and crops.

**Figure 1:**
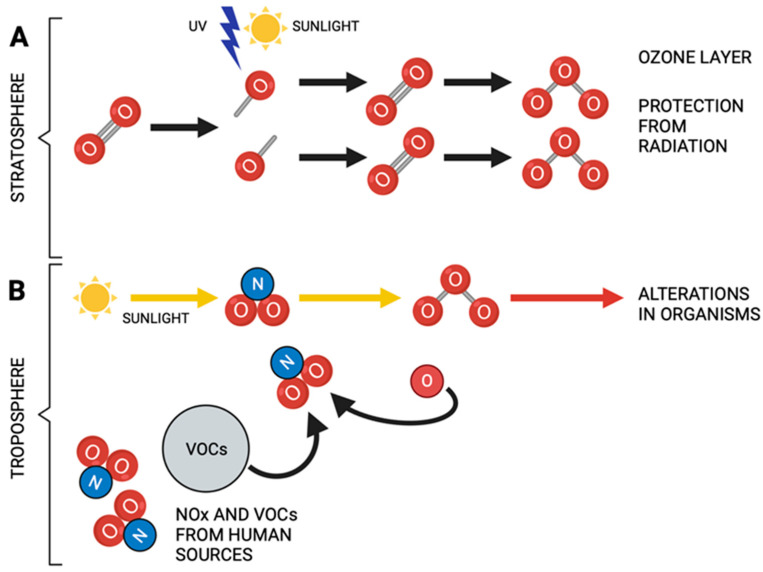
(**A**) Ozone plays a protective role in the stratosphere since it prevents the excessive passage of ultraviolet radiation to the earth. (**B**) Ozone acts as an atmospheric pollutant in the troposphere. Note the different functions of ozone when it is formed. Yellow arrows indicate ozone production from nitrogen species and solar radiation. Created in BioRender.com (accessed on 6 June 2023).

The O_3_ molecular conformation is highly reactive and has oxidizing properties. When O_3_ is inhaled, it alters the redox balance in the body. O_3_ gives rise to reactive oxygen species, such as superoxide anion (O_2_^•−^), hydrogen peroxide (H_2_O_2_), hydroxyl radical (^•^OH), nitric oxide (^•^NO), etc. The increase in these species induces changes in cell signaling, which lead to a loss of regulation of the inflammatory response and maintain a chronic state of oxidative stress. Biological systems must maintain the oxidation–reduction balance, since a large part of cell signaling depends on redox signals. The loss of oxidation–reduction balance in the organism, caused by excess oxidants, produces a state of oxidative stress. When chronic, this state plays an essential role in developing degenerative diseases, such as autoimmune diseases, cancer, heart disease, and diabetes. It also plays a crucial role in developing neurodegenerative immune disorders such as Alzheimer’s disease (AD) [5], Parkinson’s disease (PD) [7], Huntington’s disease [8], amyotrophic lateral sclerosis [9], and other processes related to pathological aging [10] (Figure 1).

## 2. Environmental Pollution and Intestine

Mucous membranes are body barriers that are in contact with the environment and constitute different habitats where microbes interact with the host. The digestive tract, respiratory tract, genitourinary tract, and skin are sites where the association with these microorganisms is essential [11]. The largest population of these microbes is found in the gastrointestinal tract, where one of their functions is to make digested food and absorbed water, which is absorbed into the body [12,13]. Moreover, the gut houses most of the mucosa-associated lymphoid tissue in the human body. The intestinal mucosa contains more lymphocytes than any other lymphatic organ [14]. There is a mutualistic relationship between the gut microbiota and the human host; the intestine provides a niche for microbes to inhabit, while the host benefits from an increase in digestive capacity favored by the microbiota and the generation of metabolites that stimulate the immune system; they also occupy a space within the intestine that prevents the accumulation of potentially pathogenic organisms [15,16]. In humans, environmental factors such as diet, pollutants, antibiotic treatments, and stress can change the composition of the gut microbiota towards a decrease in the abundance of beneficial bacterial species, accompanied by the growth of pathogenic species [16]. The disturbance in microbial structure and function is known as dysbiosis, which disrupts immune and tissue homeostasis and is associated with various inflammatory diseases inside and outside the gastrointestinal tract [17]. There are bacterial species of microbiota in the intestine that can counteract systemic inflammation. The gut microbiota is essential for host digestion and nutrition, as it can generate nutrients from substrates that the host could not otherwise digest. Intestinal microorganisms release short-chain fatty acids (SCFAs) from indigestible dietary fibers, an important energy source for intestinal mucosal cells that is essential for modulating immune responses emanating from the intestine [17]. Therefore, gut bacteria help protect against pathogenic infections through competition, secretion of antimicrobial peptides, stimulation of innate immune cells, development of lymphoid tissue, antibody production, and T-cell differentiation [18]. The gut microbiome has been shown to play a critical role in mediating immune and anti-inflammatory responses at distant sites by releasing and moving microbial metabolites such as SCFAs, cytokines, and hormones into the bloodstream [16]. The gut mucosa and associated immune system are primed to interact with food, microbiota, potentially harmful antigens, and inflammatory signals from other organs to maintain a fine homeostatic balance and promote an appropriate response, as well as to ensure the presence of harmless microbes [14,19].

**Figure 2:**
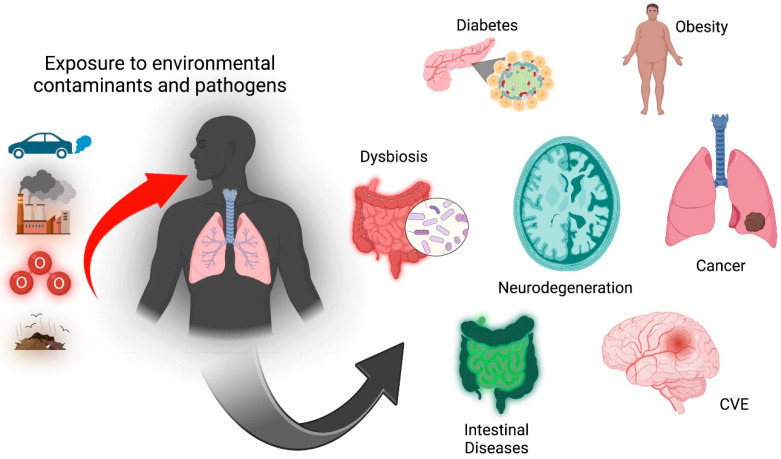
Environmental pollution is associated with developing various pathologies such as diabetes, obesity, neurodegenerative diseases (Parkinson’s, Alzheimer’s), cerebral vascular events (CVE), some types of cancer, and intestinal alterations as dysbiosis and intestinal diseases. Created in BioRender.com (accessed on 6 June 2023).

The intestine is exposed to air pollutants through direct or indirect routes. In a gas exchange in the lungs, during exhalation, the lung mucus layer can reach the oropharynx and be swallowed and enter the intestinal tract, while another mechanism by which contaminants have access to the gastrointestinal tract is through direct ingestion of food and water that contain air pollutants [20]. Blood flow is important for distributing reactive oxygen species generated in the lungs, secondary to ozone exposure, since the gastrointestinal tract is very susceptible to tobacco smoke and exposure to air pollution, contaminants which exacerbate systemic inflammation and cause oxidative damage to gut mucosa and microbiota [21]. Alterations in intestinal bacterial species, populations, and microbial metabolites are linked to changes in immune responses and inflammation in different organs [22]. Evidence obtained in recent decades strongly suggests that air pollutants, including ozone, generate adverse effects on the digestive tract and that they participate in the development of Inflammatory Bowel Disease (IBD), Crohn’s Disease (CD), appendicitis and Irritable Bowel Syndrome (IBS) [23,24], immune diseases, and chronic degenerative and neurodegenerative diseases such as Parkinson’s and Alzheimer’s disease. During the pathophysiology of these diseases, a proinflammatory environment is generated that affects the rest of the organism (Figure 2).

## 3. Intestinal Alterations and Neurodegeneration

The architecture of the intestinal epithelium is very complex; it is made up of a monolayer of cells connected by different cell junctions, such as tight junctions, gap junctions, and anchor junctions.

**Figure 3:**
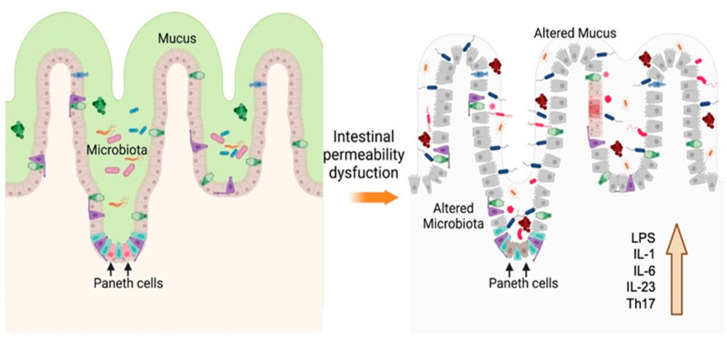
Epithelial architecture. Maintaining the intestinal barrier maintains the mucosa and the microenvironment of the microbiota. Paneth cells synthesize antimicrobial molecules that control the growth of pathogenic microorganisms. When the functioning of these cells, vesicles they produce and other factors are altered, intestinal permeability is lost, increasing the response of Th17 cells, generating an increase in IL-1β, IL-6, and LPS, among others, and establishing an inflammatory process. Created in BioRender.com (accessed on 6 June 2023).

The surface of these cells is covered by mucosa, which separates the internal environment from the external one, blocking the passage of substances, which makes it very selective. One of the main functions of these cells is the absorption of nutrients and some electrolytes, which is why it is considered a highly communicated and coordinated semi-permeable barrier to prevent the free passage of certain toxins, antigens, and microbial products. In this way, a balance is generated between these transport mechanisms, the immune system, the metabolite products of the diet, and the intestinal microbiota. An alteration in this dynamic communication would cause a loss in this intestinal barrier, which would generate an imbalance and, therefore, the free passage of some substances and the development of various immune responses [25]. This occurs in some diseases, such as IBD, UC, and CD, whose main characteristics are the presence of a chronic inflammatory process, in which the immune response plays a fundamental role as part of the etiopathogenesis of these diseases, especially the response of T lymphocytes helper 17 (Th17) [26,27] and autophagy processes. It is known that patients with IBD have altered levels of cytokines, so researchers have focused on finding an association between polymorphisms of these cytokines and the relationship between promoters such as tumor necrosis factor-alpha (TNFα) and interleukin 10 (IL-10), as well as the relationships found between IL-1 and its receptor or between the IL-6 and the IL-23 receptor [28]. It has been described that the function of Th17 is to protect the intestinal mucosa and maintain the immune microenvironment, in addition to increasing the inflammatory response through proinflammatory cytokines [29]; this function is maintained by IL-23, since it acts on the innate response and facilitates the maintenance of Th17 cells [30]. The cellular organization of the intestine that allows permeability maintenance can be compromised and facilitate the development of certain diseases. Proteins associated with granule exocytosis pathways in Paneth cells [31] and by transcription factors, such as XBP1, are known to respond to endoplasmic reticulum stress and are required for the maintenance of the secretory cells [32]. Both mechanisms generate more susceptibility to intestinal inflammation, generating the loss of intestinal permeability (Figure 3).

## 4. Intestinal Permeability and Neurodegeneration

Paneth cells, participating in granule exocytosis pathways, produce antimicrobial peptides that control populations of bacteria harmful to the intestinal microbiota [33]. There is evidence that endotoxins, which are lipopolysaccharides (LPS) and components of the cell wall of Gram-negative bacteria, generate a proinflammatory response mediated by TNFα, IL-1β, or NO (Figure 3) [34]. In a mouse model, inhalation of low levels of endotoxin after exposure to ozone promoted an increase in various factors such as IL-1β, IL-1α, and IL-6, potentiating lung lesions in mice [35]. In addition, in clinical studies, Angelico Mendy et al., 2019 concluded a synergistic association between co-exposure to endotoxin and environmental contamination, increasing the recurrence of hospitalization due to asthma attacks in children [36].

Recent studies point to the relationship between intestinal microbiota and some neurodegenerative diseases. The brain–gut-microbiota axis is a bidirectional system that allows metabolic, immune, endocrine, and neuronal signaling [37] in both directions. Some evidence links endotoxins directly to neurodegeneration through systemic inflammation and microglia activation. Systemic LPS promotes IL-1β activation of microglia, chemokines, IL-6, and acute cognitive loss in mice [38]. LPS injected intraperitoneally promotes the accumulation of Aβ 1-42 in the hippocampus and cerebral cortex in mice, generating neuronal pyroptosis [39], hyperphosphorylation of the tau protein [40], and an increase in α-synuclein [41]. On the other hand, Alzheimer’s disease has been characterized mainly as a disease in which extracellular deposits of the β-amyloid (Aβ) protein form neuritic plaques, which eventually lead to the intracellular accumulation of abnormal tau proteins and the subsequent formation of neurofibrillary tangles [42,43]. Molecular mimicry is a concept that allows us to explain how certain similarities occur in the tertiary structure of an exogenous and pathogenic protein and some human proteins [44]. This hypothesis reminds us of the formation of prions in the central nervous system and the generation of certain diseases. Particularly in Parkinson’s disease, patients have a recurrent history of gastrointestinal dysfunctions before diagnosis [45]. Various studies have generated the “seed” of α-synuclein protein that recruits other endogenous α-synuclein proteins and subsequently becomes insoluble, hyperphosphorylated, and ubiquitinated pathological proteins [46]. Different models of α-synuclein “seeding” have demonstrated the induction of pathological α-synuclein from the peripheral nervous system to the central nervous system following the injection of intramuscular α-synuclein in transgenic mice [47].

Some studies show how some species of bacteria produce extracellular amyloid protein fibers, which are helpful for environmental protection and surface adherence [48]. This protein, formed from the CsgG protein, allows a flow of macromolecules, since it forms a functional nanopore [49]. Through a complex transport system, this system creates a “curli” fiber within the system [50]. Cellular pathways activated by bacterial amyloid in neurodegeneration processes include the TLR 2/1, CD14, and NFκB system [51].

Therefore, the structural complexity of the intestinal barrier is compromised when bowel permeability fails. The mechanisms underlying this condition range from a loss in the transport of granules, a decrease in the mucus layer, changes in the intestinal microbiota, and an alteration in the production of specific metabolites, such as LPS and proteins, such as α-synuclein or amyloid protein. All these changes activate signaling pathways that increase free radicals, as well as the activation of antioxidant and immune response systems, which, when they lose regulation, establish a chronic state of oxidative stress and loss of regulation of the inflammatory response, establishing a vicious circle which is present in chronic-degenerative diseases.

## 5. Parkinson’s Disease and Intestine

Parkinson’s disease (PD) is a neurodegenerative disease with a chronic state of oxidative stress and loss of regulation of the inflammatory response. These alterations give rise to oxidation of dopamine and the formation of metabolites of this neurotransmitter, such as dopamine quinones [52]. Neuromelanin is found in catecholaminergic neurons of the substantia nigra pars compacta and locus coeruleus and protects neurons from oxidation processes. Loss of neuromelanin and subsequent depigmentation of these brain regions is a hallmark of PD. There is evidence to suggest that neuromelanin-abundant substantia nigra pars compacta dopaminergic neurons are more sensitive to cell death [53,54]. In addition, it is known that chronic low doses of O_3_ exposure produces an oxidative stress state in substantia nigra, which is accompanied by inflammatory processes and the translocation of cytochrome C to the neuron nucleus, which leads to the activation of cell death [55]. It has been proposed that there is a bidirectional communication of the brain–gut axis, since there is evidence of accumulation of α-synuclein outside the brain. The abnormal accumulation of α-synuclein in the esophagus and the submucosal in the myenteric plexus and nerve fibers in the enteric nervous system have also been demonstrated [56].

**Figure 4:**
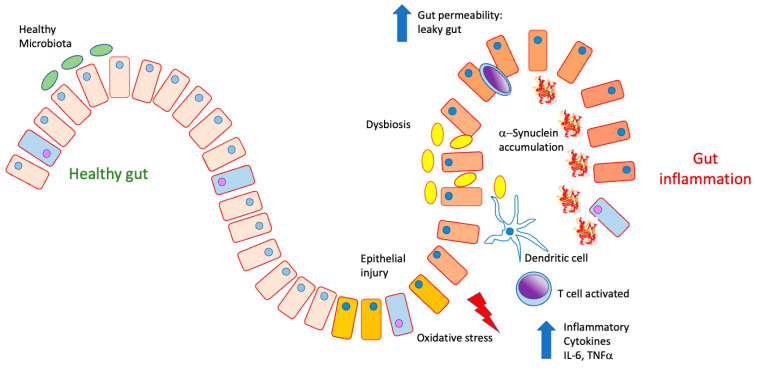
In a physiological state, the intestine presents a healthy microbiota. It can prevent food antigens and inflammatory cytokines such as TNFα, IL-6, and IL-17 from being released into the bloodstream. Under conditions of inflammation and dysbiosis due to the proliferation of pathological microbiota, the epithelial barrier is lost, increasing permeability to both pathogens and antigens, which produces inflammatory activation, the release of cytokines, and a state of oxidative stress, as well as their accumulation.

Alterations in gastrointestinal tract have been suggested as preceding changes in the brain; among these are dysphagia, increased salivation, delayed gastric emptying, and constipation [57,58], along with alterations in the microbiota and abnormal accumulation of α-synuclein in the gastrointestinal system. The intestine has an essential role as a barrier [59], since it prevents the release of food antigens and inflammatory bacteria, preventing them from entering the circulation; however, when the barrier is altered, it becomes very permeable, releasing it into the torrent blood proinflammatory molecules [60], which causes the lymphoid follicles of the mucosa to become activated; T and B lymphocytes found in Peyer’s patches adjacent to M cells proliferate as a process of stimulation by a specific antigen, releasing into the bloodstream. From there, they migrate to the lamina propria, and this is where the lymphocytes B are transformed into plasma cells, which produce specific secretory IgA against different antigens. IgA produced in the mucosa does not activate the complement pathway and does not cause inflammation [61]. In addition, an inflammatory process is induced when foods rich in antigens from pathogenic organisms such as bacteria are ingested. Matthew Stephens and Pierre-Yves von der Weid (2020) demonstrated that LPS from specific species of the bacteria *Serratia marcescens*, *Pseudomonas aeruginosa*, *Klebsiella pnemoniae*, and *Salmonella enterica* could differentially modulate TNFα through factor activation—core kB (NFkB) [62]. The intestine can distinguish between its own microbiota and pathogenic organisms. It is known that alterations between resident and pathogenic bacteria lead to an immune imbalance producing inflammation. It is accompanied by a loss of control of intestinal permeability that allows the release of bacterial LPS that reach the enteric nervous system, maintaining an inflammatory state, as occurs in CD and PD. Otherwise, it has been observed that patients with Parkinson’s disease show increased permeability in the colon (Figure 4), contributing to the systemic inflammatory process in patients with said disease.

## 6. Alzheimer’s Disease and Intestine

One of the characteristics of Alzheimer’s disease is the extracellular accumulation of β-amyloid peptide (βA)—mainly the βA1-42 isoform—which forms plaques in various regions of the cerebral cortex and intracellular neurofibrillary tangles of hyperphosphorylated tau protein in the hippocampus, medial temporal lobe, isocortical temporal, parietal areas, and frontal lobes, producing loss of regulation of neuronal metabolism such as glutamatergic, cholinergic, and GABAergic neurotransmission [63]. Oxidative stress causes a change in the conformation of this peptide, making it more insoluble and impossible to eliminate [64]. The decrease in its clearance of βA leads to the loss of neuronal homeostasis [65]. However, one of the roles that βA plays in “normal” functioning in the brain is associated with glucose metabolism, specifically in the internalization of glucose [66]. Glucose uptake in neurons is mainly carried out by the glucose transporter 3 (GLUT3) [67]. In regions such as the hippocampus, one of the main areas compromised in AD, transport is carried out through the expression of GLUT4 in neurons by binding insulin or βA1-40 to the insulin receptor, which activates the PI3K/Akt pathway, whose anti-apoptotic processes promote neuronal survival, as well as receptor translocation [66]. The pathogenesis associated with AD involves decreased hippocampal volume, cerebral hypoxia, neuroinflammation, and neuronal ischemic events, which are associated with the gradual loss of episodic memory in patients [65]. It has been observed that the oxidative damage generated by the increase in ROS due to exposure to O_3_ influences the density and functionality of neurons and glia in the hippocampus, reflecting the loss of synapses and, with it, a deficit in the functionality of the hippocampus, which could be reflected in the decrease in attention or short-term memory [68,69,70]. Studies of rats exposed to O_3_ found that the increase in ROS is directly related to the rise in the levels of peroxidized lipids and proteins, which promote apoptosis in the hippocampus [71]; specifically this occurs in the CA1, CA3, and dentate gyrus regions, increasing the production and accumulation of the βA1-42 peptide in cells in the dentate gyrus [72].

The increase in lipids and proteins oxidized by ROS in these hippocampal regions promotes the expression of βA1-42, which results from the oxidation of the sulfhydryl radical of βA1-40—specifically, methionine at position 35. This promotes a more significant interaction between βA and the unsaturation of the lipid chains of the membrane of neurons; initiates lipid peroxidation; and generates highly reactive products such as 4-hydroxy-2-nonenal (4HNE) and acrolein, which amplifies the oxidative effect of βA1-42 [73]. This causes accumulation of βA1-42, generating oligomers, which, when bound to IR, prevent its phosphorylation, decrease glucose metabolism, and inactivate the PI3K/Akt pathway, compromising survival neuron of the hippocampus [66].

**Figure 5:**
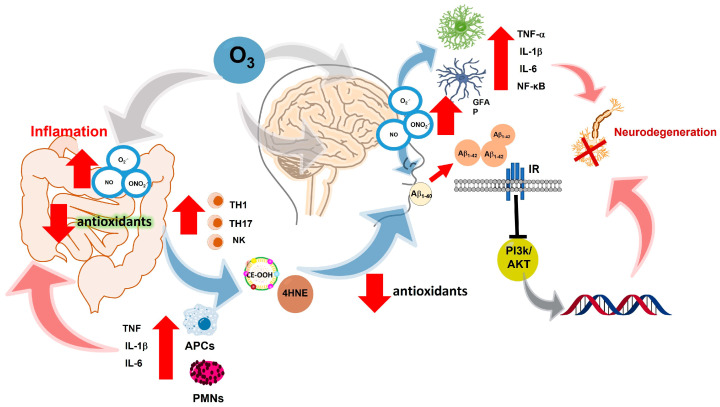
Exposure to O_3_ increases the production of ROS, which leads to the development of inflammatory bowel diseases associated with the increase of cytokines IL-6, IL-1β, and TNFα, as well as the activation of TH1, TH17, and NK cells responsible for cell maintenance; however, the increase in ROS, as well as the decrease in antioxidant defenses, limits the function of lymphocyte cells, generating peroxidized lipid adducts such as CE-OOH and 4HNE at the systemic level. This decreases antioxidant defenses and causes polypeptides such as βA to oxidize and generating oligomers of βA1-42, which interrupt the internalization of glucose. Survival pathways such as PI3K/AKT are then inhibited, causing neuronal cells to atrophy, which causes astrocytes and microglia to be activated. Proinflammatory cytokines causing neuroinflammation are released, which chronically leads to neurodegeneration of neurons in the hippocampus and cortical areas that is pathologically associated with gradual loss of memory. It is currently believed that discrete degenerative processes at the beginning of AD such as the deposition of highly insoluble βA and the accumulation of tau proteins-damage neurons, provide clear inflammatory stimuli to local microglia and the hippocampus.

Besides this, they try to eliminate these βA adducts, astrocytes, and microglia by binding to βA activate the MyD88 pathway through their toll-like 4 receptors (TLR4). This promotes the expression of the receptor for tumor necrosis factor associated with factor 6 (TRAF6) and the CD33 receptor in the hippocampus and the cerebral cortex; causes neuroinflammation due to overexpression of glial fibrillary acidic protein (GFAP); and generates astrogliosis [74,75] as well as an increase in NFkB. This promotes the expression of proinflammatory cytokines such as IL-1β, IL-6, and TNFα, atrophying hippocampal communication between neurons and the glia [71] (Figure 5).

In murine models, it has been shown that exposure to O_3_ promotes the production of NO, and this, in the presence of superoxide ion, generates the peroxynitrite ion (ONOO-). Together, these highly oxidizing species induce neurotoxicity in the hippocampus and cortical areas. The generation of NO in microglia causes high oxidation of lipids, proteins, and genetic material that favors the activation of different signaling pathways, such as the Janus kinase complex (JAK), protein tyrosine kinase (TK), protein kinase C (PKC), mitogen-activated protein kinases (MAPK), as well as NFκB and activator protein 1 (AP-1), prooxidant enzymes such as inducible nitric oxide synthase (iNOS), cyclooxygenase 2 (COX-2), and lipoxygenase 5 (5-LOX-5) [76]. In addition to the activation of these pathways, there is also an expression of proinflammatory cytokines, such as IL-6, IL-1α, IL1β, TNFα factor, and granulocyte-macrophage colony-stimulating factor, that stimulate a state of neuronal stress. This state is mediated by high levels of ROS and inflammatory mediators, resulting in neuronal death and expression of AD pathogenesis [65,76]. RAMAN spectrometry studies show that repeated exposure to O_3_ causes conformational changes in the secondary amino acids of the βA1-40 peptide, taking them from an alpha helix conformation to a beta-sheet structure, which is insoluble and impossible to eliminate [64]. Therefore, in urbanized cities such as ours, exposure to O_3_ from our childhood can affect our learning and even be a promoter of the oxidation of proteins, lipids, and genetic material that, in our adult life, are related to the overexpression of the βA1-42 peptide and neurofibrillary tangles that could lead to the development of Alzheimer’s disease.

Additionally, an increase in cholesterol ester hydroperoxides (CE-OOH) has been observed in patients with AD. These hydroperoxides are products of lipoprotein oxidation, such as chylomicrons, high-density lipoproteins (HDL), intermediate-density lipoproteins (IDL), low-density lipoproteins (LDL), and very-low-density lipoproteins (VLDL). This specifically occurs in those whose structure includes apolipoproteins AI, B48/B100, D, E, and J/clusterin, which participate in lipoprotein binding to the anchoring sites associated with ATP, in lipid transport, in cholesterol metabolism, as well as defense against oxidative damage. This increases their levels in plasma and promotes a state of oxidized hypercholesterolemia [77], which increases the expression of proinflammatory cytokines as well as inducing cell damage. Due to this increase in peroxidized lipids, the redox balance is compromised. Therefore, antioxidant proteins such as paraoxonases (PON) protect lipoproteins from oxidative stress, reducing the damage caused by ROS [78]. However, it has been observed that in patients and mouse models of AD, PON1, and PON3, located in the systemic circulation, and PON2, located throughout the intestinal tract from the duodenum to the distal colon, different subcellular compartments such as mitochondria, lysosomes, and microsomes decrease their activity, promoting intestinal inflammation [79]. Therefore, in urbanized cities such as ours, exposure to O_3_ from our childhood can have repercussions on our learning and even be a promoter of the oxidation of genetic material, proteins, and lipids, as well as the decrease in our antioxidant proteins that protect us from damage associated with oxidation; in our adult life, this damage can be related to intestinal problems associated with an increase in plasma cholesterol and the overexpression of the βA1-42 peptide and neurofibrillary tangles that could lead to the development of AD.

## 7. Discussion

It has been widely demonstrated that environmental pollution produces a chronic state of oxidative stress. Increase in ROS secondary to pollutant inhalation leads to the loss of regulation of cell functions, particularly antioxidant systems. The oxidation–reduction processes that regulate cell activity are one of the first signals to appear during evolution. Therefore, they are central to the physiological organization that allows proper cell function. ROS regulate many cellular signals (metabolic, endocrine, reproductive, etc.), particularly of the immune system. The inflammatory response is key in cell defense and repair; it is a self-limited response modulated by redox signaling. Hence, the redox balance is necessary for the immune response to maintain its function. However, repeated exposure to environmental pollution by ozone (at low doses) causes a slight increase in ROS, and the antioxidant defenses do not respond adequately. The gradual increase in ROS and the inability of the antioxidant systems induce a chronic state of oxidative stress that leads to a loss of regulation of the inflammatory response, as occurs in non-infectious chronic-degenerative diseases. The chronic state of oxidative stress is an epiphenomenon that affects the entire organism. However, depending on the antioxidant capacity and other factors, including the genetic or previous presence of disease, the target organ will be more compromised than the others [80,81]. There is a clear association between the gastrointestinal system and neurodegenerative diseases such as Parkinson’s disease and Alzheimer’s disease. There are also intrinsic alterations of the intestine in the face of oxidative stress caused by environmental pollution by ozone. The response of the duodenum and colon seems to be important for gastrointestinal pathologies, which can be explained as follows:

(1) Oxidative stress causes alterations in the intestinal wall, altering permeability and allowing the passage of high molecular weight molecules that will ultimately stimulate the immune system, causing inflammation and disease.
(2) Oxidative stress acts on the intestinal microbiome, changing its balance and favoring microorganisms whose metabolisms cause toxic molecules that contribute to further increased inflammation, forming a vicious circle between loss of regulation of the inflammatory response. It also increases intestinal permeability, causes harmful changes of intestinal microorganisms, and causes alterations of the immune response.

The association between gastrointestinal alterations and non-communicable chronic degenerative diseases shares a series of characteristics, such as the chronic state of oxidative stress, loss of regulation of the immune response, alteration in permeability of both the blood–brain and intestinal barriers. This allows passage of high molecular weight substances, formation of toxic metabolites both by the intestinal bacteria as well ROS and accumulation of insoluble proteins such as α-synuclein, amyloid peptides, etc.

Additionally, reactive species activate multiple signaling pathways; therefore, the possibility of increasing endogenous antioxidants and the consumption of probiotics and prebiotics have shown that they can be essential options in the treatment of chronic-degenerative diseases.

## 8. Conclusions

Environmental pollution by ozone generates a chronic state of oxidative stress that alters the permeability of the blood-brain barrier. The chronic state of oxidative stress is related to the loss of regulation of the inflammatory response; these two factors form a vicious circle that maintains and increases the deterioration of the degenerative disease over time.

Environmental pollution by ozone causes alterations in the intestine, inducing an inflammatory response in the intestinal wall that alters its permeability and the bacterial populations. The passage of macromolecules into the blood stimulates the immune system. The pathological change of the intestinal microbiome contributes to the production of toxic metabolites that increase the degenerative response. Loss of the permeability of the blood–brain barrier increases inflammation and, consequently, oxidative stress in the brain. Communication through neurotransmitters, hormones, and proinflammatory metabolites in the blood leads to a close interrelationship between these organs, promoting chronic-degenerative diseases.

## Data Availability

Data reported in this paper are available on request from the corresponding author.
